# Religious switching and mental disorders in young adulthood: evidence from Finnish population register data

**DOI:** 10.1093/aje/kwaf245

**Published:** 2025-11-07

**Authors:** Kaarina Reini, Martin Kolk, Jan Saarela

**Affiliations:** Demography Unit, Åbo Akademi University, 65100 Vaasa, Finland; Demography Unit, Åbo Akademi University, 65100 Vaasa, Finland; Demography Unit, Department of Sociology, Stockholm University, SE-106 91 Stockholm, Sweden; Institute for Future studies, SE-101 31 Stockholm, Sweden; Demography Unit, Åbo Akademi University, 65100 Vaasa, Finland

**Keywords:** religious switching, mental disorders, sickness absence, population registers, Finland

## Abstract

This is the first study to analyze religious switching and sickness allowance (SA) due to mental disorders using register data with ICD-codes. We performed a prospective cohort study based on population born in Finland in 1986-2003 (*n* = 1 060 280). Each person was observed from age 18 in 2004-2023. Cox proportional hazards models with and without sibling fixed effects, and logistic regression models restricted to individuals who had switched religion, were applied. We observed a 44% higher hazard of SA receipt of non-affiliated individuals, and a 27% higher hazard for those with any other religion, as compared to the majority group of Lutherans at a time point when they had not switched religion. Religious switching was associated with a 38%-118% higher hazard of SA receipt, depending on the type of switch. Results from models with and without sibling fixed effects were similar. The conditional logistic regressions revealed that, the increase in mental health-related SA receipt was steeper before the switch as compared to after the switch. However, the incidence of mental health problems remained elevated after religious switching. These findings suggest that religious switching and poor mental health are interrelated, and that the direction of causality may run in both directions.

## Introduction

Declining mental health among the young has been viewed as a defining societal challenge of our time. In 2019, 293 million of the 2.5 billion individuals aged 5 to 24 worldwide had at least one mental disorder, while 31 million had a substance use disorder.^1^ At the same time, religious affiliation patterns are shifting, particularly among younger generations. In the United States, 43% of young adults aged 18-24 identify as religiously unaffiliated, compared to just 13% of those aged 74 and older.^2^ Additionally, the proportion of Americans identifying with a religion other than Christianity has been steadily increasing and is now reaching over 7%. These parallel societal trends have led to an increasing focus on a possible linkage between religious disaffiliation and mental health.

The interrelation between mental health and religious switching is largely unexplored, with existing studies relying on self-reported health data from the General Social Survey in the United States.^3^^,^^4^ Switching from theologically, socially and culturally exclusive groups, “high-cost religion groups,” like Mormons and Jehovah’s Witnesses, is linked to poorer self-reported health.^3^ Religious disaffiliation (leaving religion for no religious affiliation) has been found related to poorer experienced health and lower well-being as compared to being consistently affiliated or consistently unaffiliated.^4^ The interrelation was strongest for Evangelical Protestants and Catholics and did not exist for mainline Protestants. Furthermore, it was found that being raised with no religion and affiliating in adulthood was associated with poorer health and well-being than being consistently unaffiliated. The disadvantages were interpreted as largely reflecting the loss of social benefits of religion that followed disaffiliation. One possible pathway that connects the loss of social support to poorer health and well-being may therefore be a reduction of resources for emotional coping. Some religions have strict guidelines for health-related behaviors and lifestyles, from which disaffiliates may no longer benefit.

There is a gap in the literature on religious switching and mental health among young people. This group is especially interesting, because adolescence and young adulthood is a period of religious conversion and movement away from religion.^5^ Since changes in family structure may be a precursor of religious switching, youths who switch religious status may have experienced events that can challenge a successful transition to adulthood^6^ and thus experience also mental health problems.^7^ In younger adulthood, religious involvement has been linked to better health behaviors,^8^ social support,^9^ and less substance use.^10^

In this paper, we focus on Finland, a country that like many other northwestern European countries is dominated by a historic state church with high membership, though relatively low average religious beliefs (Supplementary material, Appendix S1). The church instead often serves as a traditional anchor in life, providing tradition and life cycle rites. The patterns have often been referred to as “belonging without believing.” Finland is also a good case to study religious switching due to the increasing proportion of people leaving the State Church. The number of individuals without any religious affiliation has grown rapidly.^11^ This trend is characterized by generational change, where younger birth cohorts are more likely to be unaffiliated than older cohorts.^12^

We provide important new evidence on religious switching and mental disorders. A previous study showed that, members of the Evangelical Lutheran State Church are less likely than the unaffiliated or other religious groups to receive sickness allowance for mental health problems.^13^ Our hypothesis is that there are differences in the incidence of mental disorders between young adults who switch religious affiliation and those who remain affiliated or unaffiliated, and that the direction of the switch matters for the magnitude of the association. We examine these associations with Cox regressions that observe first-time sickness allowance (SA) receipt at the time point of and/or after religious switching. To address empirical issues related to the fact that people who switch religious affiliation, and those with mental health problems, may come from different families, we incorporate sibling fixed effects, and compare with results from standard models that observe variation across families. To evaluate also whether mental health problems occur before religious switching, we conduct analyses that condition on SA receipt. These logistic regressions thus study patterns of SA receipt before and after religious switching, conditional on that individuals have switched religion.

## Method

### Study group

Our analyses include all persons born in Finland in 1986-2003, meaning that they were 18-37 years old during 2004-2023. Persons born abroad are excluded because they cannot be effectively analyzed regarding SA receipt.^14^ All data preparation and analyses are conducted within Statistics Finland’s remote online access system, with permission number TK-53-1370-17. Since all data were register-based and anonymized, there was no need to seek separate ethical approval for this study alone.

### Sickness absence

Sickness absence is measured from receipt of the SA benefit. Each person is linked to data on SA receipt from the Social Insurance Institution of Finland (KELA). Diagnosis information is available from 2004 onwards. All non-retired persons, including students, aged 16-67 years are eligible for SA, which compensates for work incapacity. The benefit is paid after a waiting period of nine working days, and the amount relates to previous income. The first nine days are usually covered by the employer. The maximum period of receipt is approximately one calendar year. If work incapacity continues thereafter, persons may apply for disability pension. A person becomes eligible for SA when a physician has stated that there is a need for sick leave that will last for at least twelve calendar days. There is no national register on shorter sickness absence spells in Finland.

### Religious denomination

The population register in Finland keeps track of each person’s religious denomination, including those without any affiliation and members of non-state church denominations. The information is collected for every individual and updated on a yearly basis, meaning that a person can change religious category over time. Our raw data contain information on about 50 different denominations, which we have aggregated into three groups: members of the Evangelical Lutheran State Church, non-affiliated persons, and members of any other denomination. The latter group, referred to as Other religions, is diverse and contain both foreign religions and more home-grown protestant revival denominations. One of the bigger revival movement in Finland, the Leastadians, operate inside the Evangelical Lutheran State Church. We describe the classification in further detail in the Supplementary material (Appendix S1).

### Cox regressions

To analyze the association between religious switching and mental health-related SA receipt, we first apply Cox proportional hazard models on data that have been split by each calendar year. We run models in which first-time SA receipt due to mental disorders (all codes in the F chapter) constitute the failure event. Each person is observed from age 18. Right-censoring occurs at death, emigration, receipt of disability pension, end of the observation period, or SA receipt due to a cause other than mental disorders. Our key explanatory variables are, first, whether the individual has the same religion as at age 17, or if they have switched religion, and if so, to what religion. We estimate the risk of SA receipt for all these groups. In a second stage, we further consider whether the switch took place the same year, the preceding year, or two or more years ago.

In addition, we apply Cox proportional hazards models with sibling fixed effects. In these models, siblings share the same baseline hazard. By comparing siblings in the same family, we effectively adjust for all time-invariant observed and unobserved factors that are shared by siblings within the same family (persons with the same mother and father). We also conduct analyses where religious switching and sex are interacted, in order to evaluate any sex differences in the interrelation between changing category on religion and SA receipt. The estimates are presented as hazard ratios (HRs) with 95% confidence intervals (CIs).

### Conditional logistic regressions

To further assess the incidence of receiving mental health-related SA receipt before and after the religious switch, we designed a time-stratified study in which only individuals who undergo a religious switch were selected. Everyone included is thus a switcher and observed at multiple points in time. The study population is smaller than the one used in the Cox regressions (see Supplementary material, Table S2). To investigate whether mental health-related SA receipt increases before or after the religious switch, we used conditional logistic regression models to estimate odds ratios (ORs) with 95% CIs. Like in the Cox regressions, we observed individuals from age 18. The observation period continues until 2023 unless emigration, disability pension receipt or death occurs earlier. The outcome of interest is SA receipt from mental disorders after age 18. The time variable is structured based on the religious switch, with the year of the switch assigned the time value zero. The time point of −5 years includes the preceding time points, while the time point of +5 years includes the subsequent time points. Standard errors account for the clustered structure of the data.

### Control variables

For both the Cox and logistic regressions, we run models without and with control variables. In the latter case, we adjust for mother tongue, sex, observation year, birth year, birth order, educational level, student status (whether currently enrolled in education), family situation, region of residence, childhood family situation (at age 15), mother’s highest education, father’s highest education, mother’s labor market status (at age 15), father’s labor market status (at age 15), mother’s income quintile (at age 15), father’s income quintile (at age 15), and whether the family lived in owner-occupied dwelling (at age 15). Variables measured at age 15, sex, birth year and birth order are time invariant. Since there can be variation between siblings also on the variables measured at age 15, they can be included also in the sibling fixed effects Cox regressions. The time-invariant variables mother tongue and parent’s highest education could not be included in these regressions. All other variables are time-varying and refer to the situation at the beginning of each calendar year.

Formation and descriptive statistics of the study populations used in the Cox and logistic regressions, respectively, are found in the Supplementary material (Tables S1-S3, Figure S1).

### Statistical software

All estimations were carried out with R version 4.2.2.

## Results

### Cox regressions

Results of the Cox regressions are summarized in Tables 1 and 2. We observe differences in the hazard of mental health-related SA receipt based on religious affiliation and religious switching. Comparisons in both Tables 1 and 2 are made to Lutheran church members at an observation year when they had not switched (HR set to 1). When comparing individuals who maintain their originally observed Lutheran religion, we find a health disadvantage for individuals who are non-affiliated (44% higher hazard of mental health related SA) or affiliated with any other religion (27% higher hazard).

**Table 1:** Hazard ratios with 95% confidence intervals for mental health-related sickness allowance receipt by religious switching, results from cox regressions.

**First religion (rows)/new religion (columns)**	**Lutheran**	**Non-affiliated**	**Other religion**
			
Total population, w/o control variables			
Lutheran	1	1.54 (1.51-1.56)	2.18 (1.91-2.48)
Non-affiliated	1.53 (1.36-1.71)	1.44 (1.40-1.47)	1.44 (1.27-1.63)
Other religion	1.38 (0.92-2.09)	1.83 (1.67-2.01)	1.27 (1.22-1.32)
			
Total population, with control variables			
Lutheran	1	1.46 (1.44-1.48)	1.86 (1.63-2.13)
Non-affiliated	1.35 (1.20-1.51)	1.32 (1.30-1.35)	1.42 (1.25-1.61)
Other religion	1.09 (0.72-1.66)	1.63 (1.49-1.79)	1.20 (1.16-1.25)
			
Sibling fixed effects, w/o control variables			
Lutheran	1	1.40 (1.37-1.44)	1.81 (1.46-2.24)
Non-affiliated	1.43 (1.17-1.75)	1.48 (1.38-1.58)	1.40 (1.13-1.73)
Other religion	1.03 (0.58-1.86)	1.62 (1.30-2.01)	1.24 (1.05-1.46)
			
Sibling fixed effects, with control variables			
Lutheran	1	1.39 (1.35-1.42)	1.72 (1.36-2.17)
Non-affiliated	1.35 (1.09-1.67)	1.41 (1.31-1.51)	1.38 (1.09-1.75)
Other religion	0.87 (0.45-1.68)	1.65 (1.29-2.11)	1.25 (1.04-1.50)

**Table 2:** Hazard ratios with 95% confidence intervals for mental health-related sickness allowance receipt by time since religious switching, results from cox regressions.

**Religion (rows)/time (columns)**	**No switch**	**Year of switch**	**1 year after switch**	**2+ years after switch**
Total population, w/o control var.				
Lutheran	1			
Lutheran to non-affiliated		1.60 (1.55-1.65)	1.60 (1.55-1.65)	1.51 (1.48-1.53)
Lutheran to other religion		2.54 (1.84-3.50)	2.10 (1.46-3.02)	2.12 (1.82-2.47)
				
Non-affiliated	1.44 (1.40-1.47)			
Non-affiliated to Lutheran		1.50 (1.09-2.05)	1.77 (1.31-2.38)	1.49 (1.30-1.70)
Non-affiliated to other religion		1.33 (0.93-1.89)	1.45 (1.03-2.04)	1.45 (1.26-1.68)
				
Other religion	1.27 (1.22-1.32)			
Other religion to Lutheran		1.30 (0.42-4.03)	2.72 (1.22-6.07)	1.15 (0.69-1.94)
Other religion to non-affiliated		1.96 (1.58-2.43)	2.09 (1.68-2.61)	1.74 (1.55-1.94)
				
Total population, with control var.				
Lutheran	1			
Lutheran to non-affiliated		1.51 (1.46-1.55)	1.52 (1.48-1.57)	1.44 (1.41-1.46)
Lutheran to Other religion		2.13 (1.54-2.94)	1.79 (1.24-2.58)	1.82 (1.56-2.13)
				
Non-affiliated	1.32 (1.30-1.35)			
Non-affiliated to Lutheran		1.31 (0.96-1.80)	1.55 (1.16-2.09)	1.31 (1.15-1.50)
Non-affiliated to other religion		1.31 (0.92-1.86)	1.45 (1.03-2.03)	1.43 (1.24-1.66)
				
Other religion	1.20 (1.16-1.25)			
Other religion to Lutheran		1.06 (0.34-3.27)	2.18 (0.97-4.88)	0.90 (0.54-1.52)
Other religion to non-affiliated		1.71 (1.38-2.12)	1.83 (1.47-2.28)	1.56 (1.40-1.75)
				
Sibling fixed effects, w/o control var.				
Lutheran	1			
Lutheran to non-affiliated		1.42 (1.36-1.48)	1.45 (1.38-1.52)	1.38 (1.34-1.42)
Lutheran to other religion		1.60 (0.95-2.70)	2.82 (1.62-4.92)	1.71 (1.32-2.20)
				
Non-affiliated	1.48 (1.38-1.58)			
Non-affiliated to Lutheran		1.29 (0.83-2.00)	1.38 (0.81-2.34)	1.49 (1.17-1.90)
Non-affiliated to other religion		1.65 (1.06-2.56)	1.40 (0.91-2.15)	1.30 (1.01-1.69)
				
Other religion	1.24 (1.05-1.46)			
Other religion to Lutheran		n.a.	3.87 (1.08-13.85)	1.18 (0.53-2.64)
Other religion to non-affiliated		1.57 (1.12-2.21)	1.81 (1.27-2.57)	1.56 (1.20-2.03)
				
Sibling fixed effects, with control var.				
Lutheran	1			
Lutheran to non-affiliated		1.41 (1.35-1.48)	1.40 (1.33-1.47)	1.38 (1.33-1.42)
Lutheran to other religion		1.62 (0.89-2.94)	2.71 (1.41-5.19)	1.60 (1.23-2.09)
				
Non-affiliated	1.41 (1.31-1.51)			
Non-affiliated to Lutheran		1.29 (0.80-2.08)	1.31 (0.75-2.28)	1.37 (1.05-1.79)
Non-affiliated to other religion		1.69 (1.10-2.59)	1.37 (0.82-2.27)	1.27 (0.94-1.71)
				
Other religion	1.25 (1.04-1.50)			
Other religion to Lutheran		n.a.	3.63 (0.73-18.11)	0.97 (0.42-2.26)
Other religion to non-affiliated		1.71 (1.15-2.54)	1.76 (1.18-2.11)	1.58 (1.18-2.11)

Switching is typically associated with an increased risk of poor mental health. Switching from being Lutheran to being non-affiliated is associated with a 54% higher hazard, and a 118% higher hazard if the switch is to any other religion, compared to not switching from being Lutheran (Table 1). Switching from non-affiliation to Lutheran is associated with a 54% higher hazard, and switching from non-affiliation to any other religion with a 44% higher hazard. Switching from any other religion to Lutheran relates to a 38% higher hazard, and switching from any other religion to non-affiliation is associated with an 83% higher hazard. The inclusion of control variables reduces the hazard ratios, particularly for switches from Lutheran to other religion, non-affiliated to Lutheran, and from other religion to Lutheran, but the overall patterns remain.

By applying sibling fixed effects, we can control for time-invariant family-related characteristics that are otherwise difficult to capture. In this sibling fixed effects design, we thus compare only siblings, and where at least one sibling has switched religion. When comparing these analyses to those conducted for the total population, similar patterns emerge. However, unobserved characteristics as reflected by the sibling fixed effects approach explain some of the differences, particularly for switching from being non-affiliated to Lutheran and from being Lutheran to other religion.

Using interactions, we analyzed also the hazards by sex, and found that there were only minor differences between men and women (Supplementary material, Tables S4 and S5). Family characteristics explained more of the difference for women who changed status from Lutheran to non-affiliation and from non-affiliation to Lutheran or any other religion, as compared to other types of switches. For men, the sibling fixed effects regressions revealed that unobserved characteristics explained more of the difference for men who switch from Lutheran to non-affiliation or other religion, as compared to other types of switches. Overall, the estimated effect sizes decreased more for women than for men when control variables were included.

Table 2 gives hazard ratios for mental health-related SA receipt by time since religious switching. The reference group is again Lutherans at a time point when they had not switched (HR set to 1). The hazard remains 51%-60% higher if switching from Lutheran to non-affiliation, and it is even slightly higher than for non-affiliated with no switch. Switching from Lutheran to other religion is associated with a 110%-154% higher hazard. Switching from non-affiliation to Lutheran is related to a hazard comparable to that if remaining non-affiliated, although it peaks in the year following the switch. Switching from non-affiliation to other religion is associated with a similar hazard as that for remaining non-affiliated. Adding control variables to the regressions reduces the hazard ratio for switching from Lutheran to other religion, from non-affiliation to Lutheran, and from other religion to Lutheran or non-affiliation.

Although not fully consistent, estimates from sibling fixed effects regressions that include control variables are generally somewhat smaller or close to those of the standard (non-fixed) regressions that include control variables. Sex-specific comparisons revealed some minor differences between women and men, but the overall conclusions were similar as in the pooled results presented in Tables 1 and 2 (Supplementary material, Tables S6 and S7).

In the Supplementary material (Tables S8 and S9), we present hazard ratios according to religious switching for age groups 18-24 years and 25-37 years separately.

### Conditional logistic regressions

**Figure 1:**
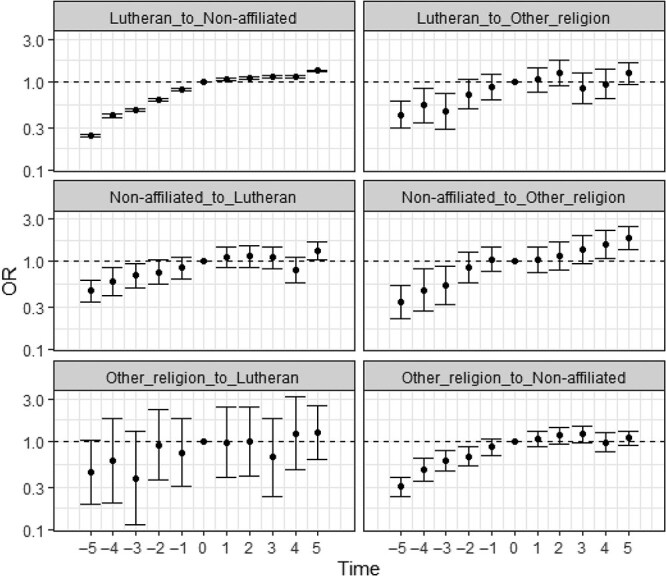
Odds ratios with 95% confidence intervals for mental health-related SA receipt according to time before and after religious switching, by type of switch, with no control variables included, results from conditional logistic regressions. Notes: Time points −5 and +5 are pooled to include all earlier and later time points, respectively. Odds ratios are presented on a logarithmic scale.

**Figure 2:**
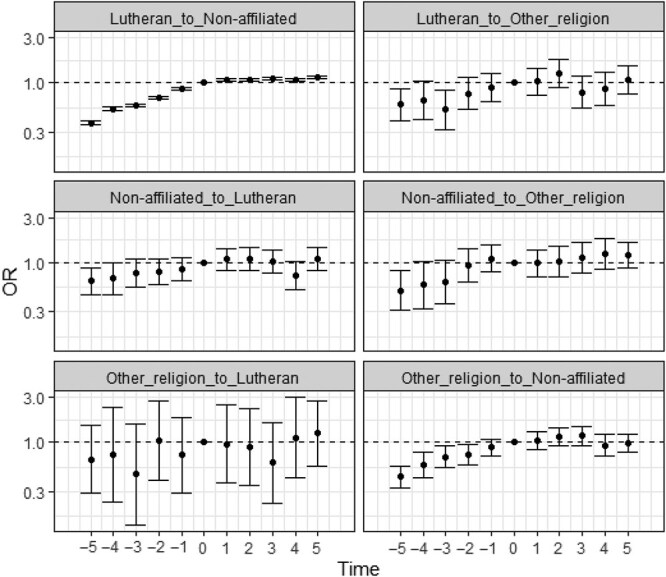
Odds ratios with 95% confidence intervals for mental health-related SA receipt according to time before and after religious switching, by type of switch, with control variables included, results from conditional logistic regressions. Notes: Time points −5 and +5 are pooled to include all earlier and later time points, respectively. Odds ratios are presented on a logarithmic scale. Control variables included are sex, mother tongue, birth order, birth year, observation year, education, family status, family situation (at age 15), mother’s highest education, father’s highest education, mother’s income quintile (at age 15), father’s income quintile (at age 15), mother’s labor market status (at age 15), father’s labor market status (at age 15), owner occupied dwelling (at age 15), student, and region of residence.

We also evaluated mental health-related SA receipt before and after switching religion. To this purpose, we restricted the study population to only individuals who had switched religious status and performed conditional logistic regressions. The results from regressions without control variables are presented in Figure 1 and those with control variables in Figure 2. Estimates from these two types of regressions are highly similar.

The odds of mental health-related SA receipt was typically found to be lower in the years preceding the religion switch as compared to the year of the switch, and in the years after the switch they were generally above that level. For instance, leaving the Lutheran church and becoming non-affiliated is associated with a 14%-63% lower odds of SA receipt before the religious switch as compared to the year of the switch. After the switch, the odds are 7%-15% higher. The pattern was similar across all six types of religious switches, but it was most pronounced for switches from Lutheran to non-affiliation, from non-affiliated to other religion, and from other religion to non-affiliated. Notably, there was a stepwise increase in SA receipt before the religious switch, and this increase was generally steeper before the switch as compared to after the switch. Mental health problems thus increase notably before religious switching and show less variation across the period after the switch. Based on models with interactions, we found that patterns for men and women were similar, although the analyses at some instances suffered from insufficient number of observations (Supplementary material, Figures S2-S6).

Results of conditional logistic regressions where we separated age groups 18-24 years and 25-37 years (Supplementary material, Figures S7-S10) resemble the above mentioned findings.

## Discussion

The interrelation between religiosity and mental health has been demonstrated in numerous studies.^15-23^ Far less explored is how mental health is affected when people switch their religion. All previous research on religious switching and mental health has been based on self-rated measures from surveys.^3^^,^^4^^,^^24^ This study provides the first register-based evidence using detailed data on religious affiliation and SA receipt due to mental disorders for the entire population born in Finland in 1986-2003.

We find that young Finns who remain Lutheran have a lower hazard compared to those who are non-affiliated or affiliate with other religions, which aligns with evidence from a recent study.^13^ Moreover, our results agree with findings from the United States.^3^^,^^4^ We see that switching from the Lutheran religion to being non-affiliated or to membership in any other religion relates to poorer mental health. Poor mental health is also more common if switching from being non-affiliated to Lutheran or any other religion. Socioeconomic and other control variables explained a notable part of the elevated risk of mental disorders when switching from being non-affiliated to Lutheran and for switching from Lutheran to other religion. The elevated hazard when switching from being non-affiliated to Lutheran, from Lutheran to other religion, and from any other religion to Lutheran was smaller also when siblings were compared.

From regressions that account for time since the religious switch, we could see that the elevated hazard remained fairly constant over time, and particularly so if switching from being Lutheran to non-affiliation. Hazard ratios were generally smaller in the sibling fixed effects regressions than in the standard (non-fixed) regressions, which suggests that unobserved heterogeneity across individuals from different families plays an important role for the interrelation between religious switching and mental disorders.

Analyses restricted to individuals who (ever) switched religion confirmed that the risk of mental health-related SA receipt was larger after the switch than in the period before it. The pattern was similar for all types of religious switches studied and for both sexes. We observed a stepwise increase in the risk of mental health-related SA prior to the religious switch, and that this increase was steeper before the switch as compared to after the switch. This is consistent with a scenario where an accumulation of psychologically challenging life events may ultimately contribute to both mental health disorders and religious switching. Switching as such seems not to reverse mental health trajectories, though it can be seen as stabilizing mental health. The link between stressful life events and mental health is well established,^25^ and many individuals experience religious or spiritual struggles following particularly stressful life events.^26^ Future studies in this field could tentatively focus on isolating such events, and how they relate to detailed mental health outcomes, such as severe mental illness, anxiety, and depression.

Previous studies on self-reported measures of religious switching and health suggest that members of high-cost religious groups and non-switchers are healthier than religious switchers.^3^^,^^4^^,^^24^ This is likely a reflection of factors that promote well-being and protect against aspects like substance use, sexual risk-taking, and depression.^27^ People who leave high-cost religious groups tend to report poorer health compared to those who leave other religious groups, which could be due to both selection and causation. Individuals with poor health may struggle to participate actively and choose to leave the group, while the decision to leave may also result in the loss of health benefits, including informal and formal networks of social support. In our data, high-cost religious groups fall under the category “other religion.” Consistent with previous research,^3^ we observe that switching from this group and to becoming non-affiliated is associated with an elevated risk of mental disorders, as compared to remaining within the group.

It is important to acknowledge that people may leave religious groups for internal issues and not solely due to mental health problems. Religious disaffiliation can bring positive experiences, such as joy, freedom, relief, gratitude, and empowerment.^28^ This is plausible if the religious group has failed to provide social support and instead has fostered feelings of judgment and isolation.

Findings from the United States^4^ indicate that the poorer health and lower well-being of religious disaffiliates, as compared to consistently affiliated and consistently unaffiliated persons, is mediated by the frequency of church attendance. Disaffiliates attend church less often, and social processes therefore seem to be important. We observe that leaving a religion to become non-affiliated is associated with a higher risk of mental disorders than not switching from a religious affiliation or if remaining non-affiliated. However, we also find that leaving the Lutheran church and becoming non-affiliated is associated with a risk of mental disorders that is comparable to remaining non-affiliated.

Religious social identity has been identified as a key factor in the relationship between religious participation and subjective psychological well-being.^29^ A strong identity, irrespective of whether it is religious or secular, have been argued as important for mental health.^24^ This would mean that also people who remain within a religious group after having considered leaving, may experience depressive symptoms, and a greater increase in symptoms over time as compared to those who never had considered leaving, and to non-religious individuals. From a public health perspective, secular alternatives to religious service attendance may help improve population mental well-being.^30^

Many previous analyses in this field have been constrained by small samples, limited follow-up, and heterogeneous study populations.^31^ A strength of this study is the use of extensive population register, which cover the total population of young adults in Finland. Register data on denomination and ICD-10-coded sickness absence offer several advantages. Mental health and religion are sensitive topics that may be left unanswered in surveys potentially biasing results. With register data we avoid selective reporting, drop-out, and memory flaws. Problems of defining the concepts of religion and mental health have been avoided, because our register-based measures can be considered objective and not self-assessed. However, they rely on that individuals communicate and interact with the public health care system and authorities in general.

A limitation of our study is that sickness allowance reflects fairly severe health problems, as it requires at least two weeks off work. Caution is needed when drawing conclusions about the mechanisms linking religious switching to mental disorders. The direction of causality may run in both directions, and our results support this interpretation. Another limitation is that the measure for religious affiliation (and non-affiliation) accounts for individuals’ congregation only, without any details on their religiosity, attendance at religious services, or religious beliefs. The register data lack information on health behaviors (such as drinking or smoking) or personality features, other than what is reflected by the control variables and what we account for with the statistical approaches. Notably, selecting “no religion” may reflect indifference or weak religious identity rather than deliberate rejection, whereas switching religion usually entails a more active, reflective decision often linked to personal conviction or life changes.

To conclude, we observed an association between religious switching and the incidence of mental health-related SA receipt. The highest incidence was found if switching from being Lutheran to any other religion. However, the incidence of mental disorders increased also before any religious switch, and it generally remained elevated thereafter. An accumulation of mental health problems and challenging life events may ultimately contribute to both mental health disorders and religious switches. Our results point to the potential protective role of stable religious affiliation and social environments, as seen in the relatively low risk of mental-health related SA receipt if remaining a member of the Evangelical Lutheran State Church. The social support and community aspects inherent in longstanding religious participation may contribute to better mental health, while disaffiliation could signify a disruption of these beneficial social networks, leading to heightened vulnerability. From the perspective of group sizes and the increasing level of secularization, people of primary interest for future studies are those who disaffiliate from the state church and those who make the reverse switch from being non-affiliated to becoming a member of the state church. Socioeconomic status and family background seem to have a notable impact on the relative mental health position of the latter group, while such characteristics appear to have only a modest impact in the former group.

## Supplementary Material

Web_Material_kwaf245

## Data Availability

The data used in the study can be obtained from Statistics Finland and Social Insurance Institution of Finland by other researchers, however, service fees apply. All data preparation and analyses were conducted within Statistics Finland’s remote online access system, with permission number TK-53-1370-17. Since all data were register-based and anonymized, there was no need to seek separate ethical approval for this study alone. As the data was analyzed anonymously there was no need to obtain consent from the study group.
