# Supplementary material for: Religious switching and mental disorders in young adulthood: evidence from Finnish population register data
Source: Am J Epidemiol. 2025 Nov 7;195(5):1276–83. doi: 10.1093/aje/kwaf245 (PMC13148995; doi:10.1093/aje/kwaf245)
Supplement: Web_Material_kwaf245 [file web_material_kwaf245.docx]

**Supplementary material for**

Religious switching and mental disorders in young adulthood: evidence from Finnish population register data

Kaarina Reini

Martin Kolk

Jan Saarela

**This PDF file includes:**

Appendix S1

Tables S1-S9

Figures S1-S10

**Appendix S1**

**Religion in Finland**

Religion in Finland is characterized by a long history of a majority Lutheran population, organized under a Lutheran state church. Religious diversity was low, until various mostly revival movements in the 19th century. In the second half of the 20th century globalization has slowly increased religious diversity in Finland.

Religion in the Nordic countries have been described as “belonging without believing”, where the church fulfils many life course rites and passages, and is closely associated with tradition, belonging, and the history of the state, while religious practices among members can be quite low (1). The nominal characteristic of the religiously affiliated should not be overstated though, as members of the Lutheran State church are still substantially more religious than the non-affiliated (2). Compared to North America, religious practices and believes are less intense, believes in key Christian tenets lower (2), and the religious landscape is much less diverse, due to the long history of state-supported Lutheranism (1).

Finland has undergone rapid secularization and is often ranked among the most secular nations in Europe (3). Membership in religious denominations has declined sharply, falling from 90% in the early 1970s to about two-thirds of the population today, while by the 2020s roughly every third Finn reported no religious affiliation (4). The country has two national churches: the Evangelical Lutheran Church of Finland, which still encompasses approximately two-thirds of the population, and the Orthodox Church of Finland, with about 1% membership. As a result, Finland’s religious landscape is characterized by a dominant Lutheran majority, a small Orthodox minority, and a substantial share of religious “nones”.

The Evangelical Lutheran Church of Finland and the Orthodox Church of Finland are equivalent to state churches in other Nordic countries, enjoying privileged status and holding important ceremonial and administrative roles, such as marriage and taxation. Because of the historical overlap between the state church and government, and the church’s traditional role in population administration, Finland has long maintained records on religious affiliation. Government registration, where individuals are registered as part of a religious denomination (including no denomination), is regulated by law. Since 2003, religious education in primary and secondary schools has been reformed into nonconfessional classes, providing general knowledge about different religions. The main reasons for leaving the Evangelical Lutheran Church include disagreements with its values and beliefs, as well as dissatisfaction with its stance on moral issues, and the avoidance of church taxes (5, 6).

Many minority religions have remained stable and have even grown somewhat in size. A growing number of minority religions reflects increasing international migration to Finland but also the potential that people with strong religious beliefs often find their identity within smaller denominations characterized by greater religiosity among their members when compared with those in the Evangelical Lutheran Church.

The levels of religiosity differ markedly between the Evangelical Lutheran Church members and the religious nones, which indicate that denominational affiliation captures a dimension of spiritual experience. On the question “How religious are you?” (on a scale from 0 to 10, with 10 being very religious) in Finnish ESS data^[[1]](#footnote-1)^, a fifth of the religious nones assign a value above 7 (2). Among the least religious denominational group (Lutherans), notably more, or half assess their level at above 7. Approximately 15% of the non-affiliated attend religious services at least once monthly, while the corresponding number is 50% among the Lutherans. Religiosity among other denominations is higher than in the Evangelical Lutheran Church, though the difference is not substantial.

**Religion in Finnish administrative data**

The data we use are administrative records of each individual’s congregation for the entire Finnish population, which has been provided by Statistics Finland. In this context, religious affiliation is a combination of official church registers and self-reported information from the denominations to the Digital and Population Data Services Agency in Finland. In Finland, all registered religious communities that have registered under the Ministry of Education and Culture have the legal obligation to annually report a list of their members to the state authority. Membership registration is crucial for the community to obtain state funding, which depends on the size of the community (4). Registration is also important for people who wish to be engaged in denomination-related activities, such as state-provided education on religious knowledge in public schools for the children. Religious affiliation has direct impact on the annual taxes individuals must pay, so it is not merely a subjective self-reported survey response. It rather has direct practical consequences, and both denominations and individuals have incentives to make sure that the information is correct.

The raw data contain about 50 different denominations. For parsimony, and because the Evangelical Lutheran Church and non-affiliated (people with no religion) constitute the largest two categories, we distinguish them, and separate them from all other denominations (other religions). Descriptive statistics on the distributions are found in Table S1. The small category of other religions consist of Orthodox Christians (the Orthodox state church plus Orthodox denominations that are not part of it), other Protestants (various Protestant churches independent of the state church), other Christians (denominations such as Jehovah’s Witnesses and the Latter Day Saints church), Catholicism, Islam, Eastern religions (various denominations, such as Buddhists, Bahai, and Hindus), and Judaism.

**References**

1. Iversen HR. Rites of ordination and commitment in the churches of the Nordic countries: Theology and terminology. Chicago, IL: Museum Tusculanum Press, 2006.
2. Kolk M, Saarela J. Religion and fertility: A longitudinal register study examining differences by sex, parity, partner’s religion, and religious conversion in Finland. Eur J Popul 2024;40(1):9. doi:10.1007/s10680-023-09693-0.
3. Voas D, Doebler S. Secularization in Europe: Religious change between and within birth cohorts. Relig Soc Cent East Eur 2011;4(1):39–62.
4. Xia W, Kolk M, Saarela J. Socioeconomic advantage or community attachment? A register-based study on the difference in national Lutheran Church affiliation between Finnish and Swedish speakers in Finland. J Sci Study Relig 2024;63:543–560. doi:10.1111/jssr.12906.
5. Äystö T, Koivula A, Wessman A, Kyyrö J, Hjelm T. Miksi suomalaiset eroavat evankelis-luterilaisesta kirkosta? [Why do Finns leave the Evangelical Lutheran Church? (In Finnish)] Yhteiskuntapolitiikka 2022;87(2):129–140.
6. Niemelä K. “No longer believing in belonging”: A longitudinal study of Finnish Generation Y from confirmation experience to church-leaving. Soc Compass 2015;62(2):172–186.

Table S1. Descriptive statistics of the study population analysed with the Cox regressions

Table S1 continued

Table S1 continued

Table S2. Descriptive statistics of the study population analysed with the conditional logistic regressions

Table S2 continued

Table S2 continued

Table S3. Descriptive statistics of the number of religion switches, (first-time) mental health related sickness allowance receipts, persons, and cumulative number of person years by age for the study population analysed in the Cox regressions

* Age groups 36 and 37 combined due to data protection regulations.

Table S4. Hazard ratios with 95% confidence intervals for mental health-related SA receipt by religious switching for men, results from Cox regressions

Table S5. Hazard ratios with 95% confidence intervals for mental health-related SA receipt by religious switching for women, results from Cox regressions

Table S6. Hazard ratios with 95% confidence intervals for mental health-related SA receipt by time since religious switching for men, results from Cox regressions

Table S7. Hazard ratios with 95% confidence intervals for mental health-related SA receipt by time since religious switching for women, results from Cox regressions

Table S8. Hazard ratios with 95% confidence intervals for mental health-related SA receipt by religious switching for 18-24 years old, results from Cox regressions


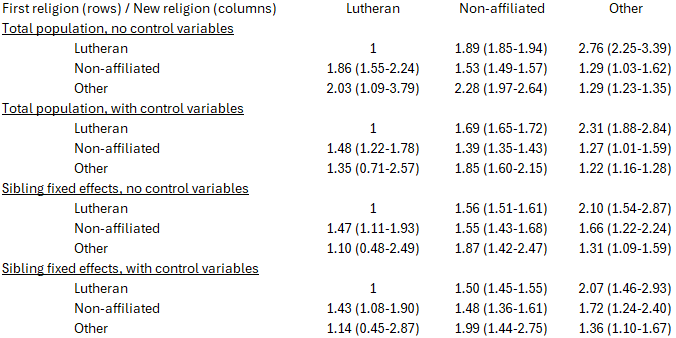


Table S9. Hazard ratios with 95% confidence intervals for mental health-related SA receipt by religious switching for 25-37 years old, results from Cox regressions


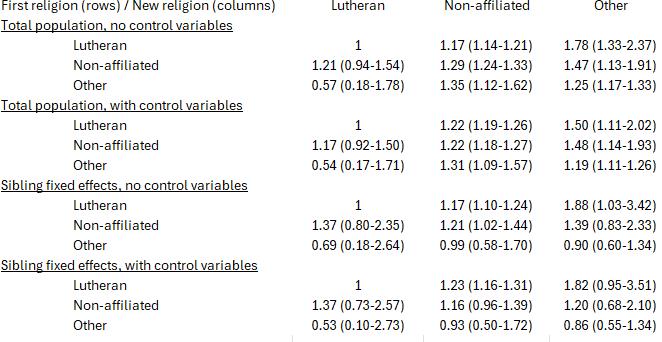


Construction of the study populations


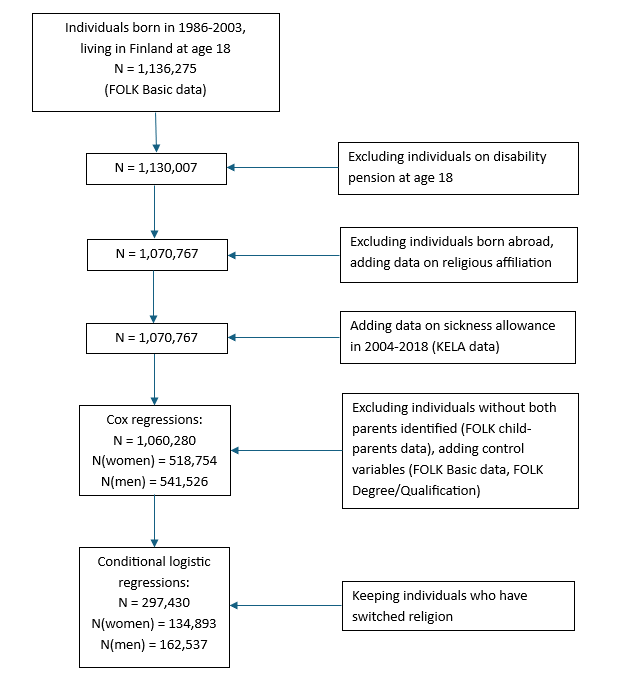


Figure S1. Description of data construction for study populations analysed with the Cox and conditional logistic regressions


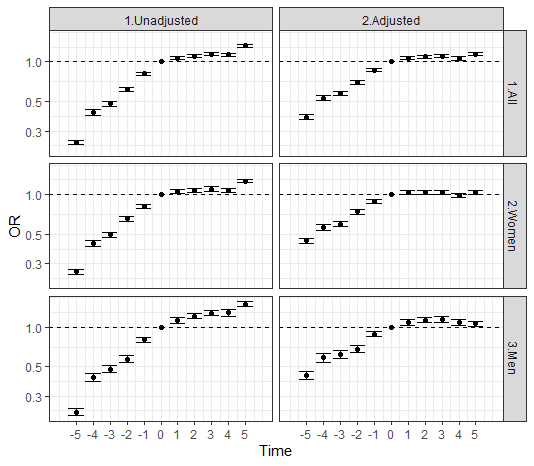


Figure S2. Odds ratios with 95% confidence intervals for mental-health related SA receipt according to time before and after any type of religious switching, for both sexes, for women and for men, without and with control variables included, results from conditional logistic regressions

Notes: Time points -5 and +5 are pooled to include all earlier and later time points, respectively. Odds ratios are presented on a logarithmic scale. Control variables included in the adjusted regression for both sexes are type of religious switch, sex, mother tongue, birth order, birth year, observation year, education, family status, family situation (at age 15), mother’s highest education, father’s highest education, mother’s income quintile (at age 15), father’s income quintile (at age 15), mother’s labour market status (at age 15), father’s labour market status (at age 15), owner occupied dwelling (at age 15), student, and region. Control variabes included in the adjusted regression for each sex are the same as above, except sex.


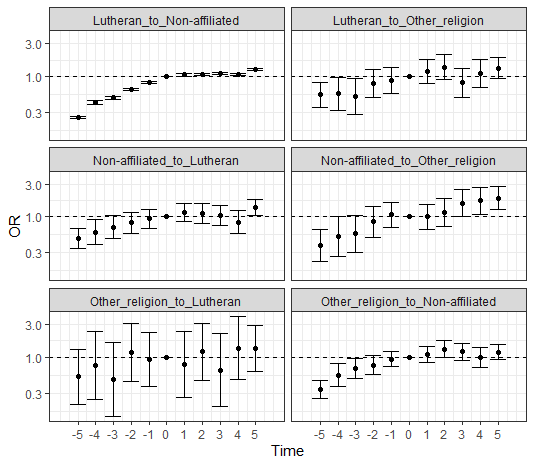


Figure S3. Odds ratios with 95% confidence intervals for mental-health related SA receipt according to time before and after religious switching, by type of switch, for women, without control variables included, results from conditional logistic regressions

Notes: Time points -5 and +5 are pooled to include all earlier and later time points, respectively. Odds ratios are presented on a logarithmic scale.


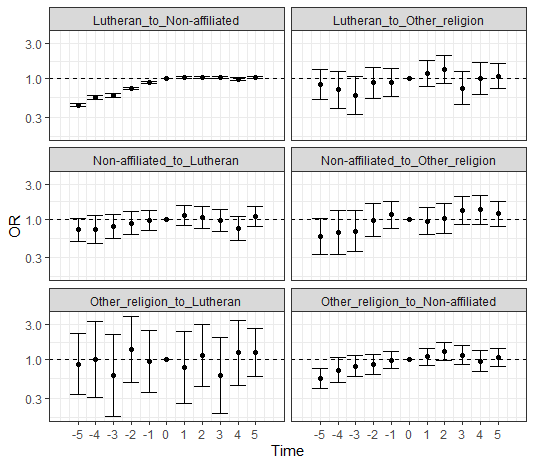


Figure S4. Odds ratios with 95% confidence intervals for mental-health related SA receipt according to time before and after religious switching, by type of switch, for women, with control variables included, results from conditional logistic regressions

Notes: Time points -5 and +5 are pooled to include all earlier and later time points, respectively. Odds ratios are presented on a logarithmic scale. Control variables included in the regressions are mother tongue, birth order, birth year, observation year, education, family status, family situation (at age 15), mother’s highest education, father’s highest education, mother’s income quintile (at age 15), father’s income quintile (at age 15), mother’s labour market status (at age 15), father’s labour market status (at age 15), owner occupied dwelling (at age 15), student, and region.


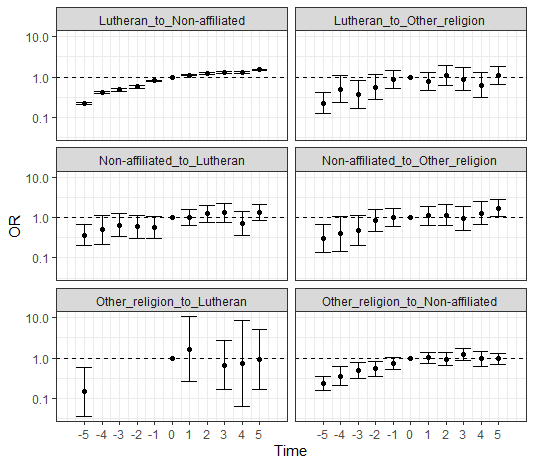


Figure S5. Odds ratios with 95% confidence intervals for mental-health related SA receipt according to time before and after religious switching, by type of switch, for men, without control variables included, results from conditional logistic regressions

Notes: Time points -5 and +5 are pooled to include all earlier and later time points, respectively. Odds ratios are presented on a logarithmic scale.


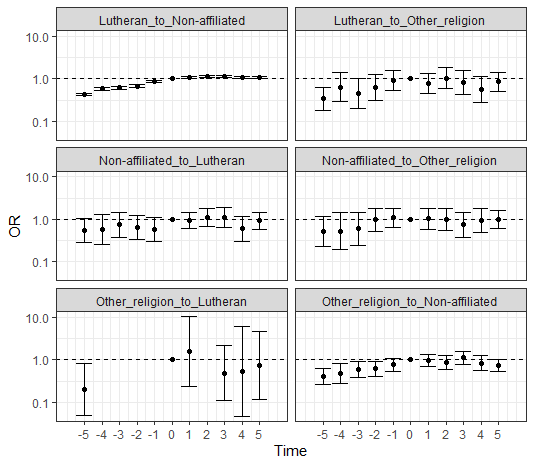


Figure S6. Odds ratios with 95% confidence intervals for mental-health related SA receipt according to time before and after religious switching, by type of switch, for men, with control variables included, results from conditional logistic regressions

Notes: Time points -5 and +5 are pooled to include all earlier and later time points, respectively. Odds ratios are presented on a logarithmic scale. Control variables included in the regressions are mother tongue, birth order, birth year, observation year, education, family status, family situation (at age 15), mother’s highest education, father’s highest education, mother’s income quintile (at age 15), father’s income quintile (at age 15), mother’s labour market status (at age 15), father’s labour market status (at age 15), owner occupied dwelling (at age 15), student, and region.


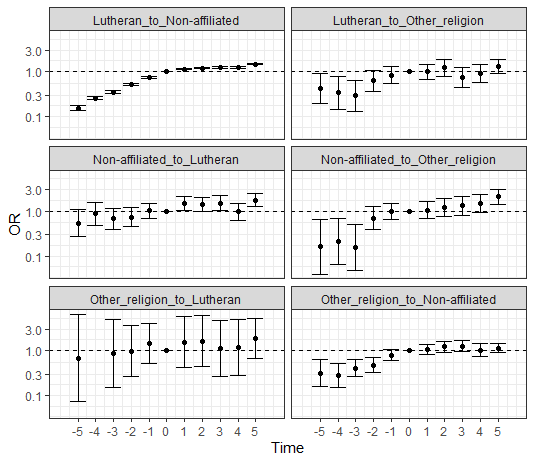


Figure S7. Odds ratios with 95% confidence intervals for mental-health related SA receipt according to time before and after religious switching, by type of switch, for age group 18-24, without control variables included, results from conditional logistic regressions

Notes: Time points -5 and +5 are pooled to include all earlier and later time points, respectively. Odds ratios are presented on a logarithmic scale.


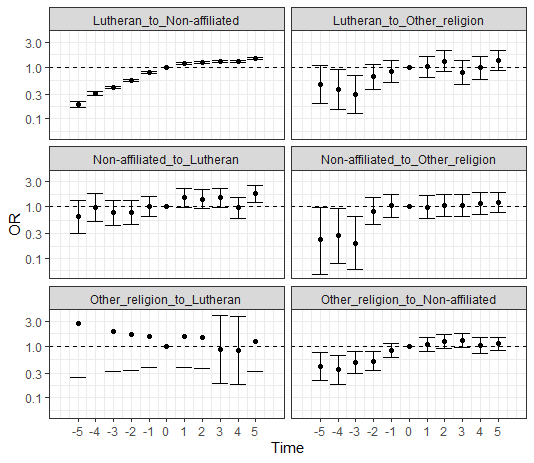


Figure S8. Odds ratios with 95% confidence intervals for mental-health related SA receipt according to time before and after religious switching, by type of switch, for age group 18-24, with control variables included, results from conditional logistic regressions

Notes: Time points -5 and +5 are pooled to include all earlier and later time points, respectively. Odds ratios are presented on a logarithmic scale. Control variables included in the regressions are mother tongue, birth order, birth year, observation year, education, family status, family situation (at age 15), mother’s highest education, father’s highest education, mother’s income quintile (at age 15), father’s income quintile (at age 15), mother’s labour market status (at age 15), father’s labour market status (at age 15), owner occupied dwelling (at age 15), student, and region.


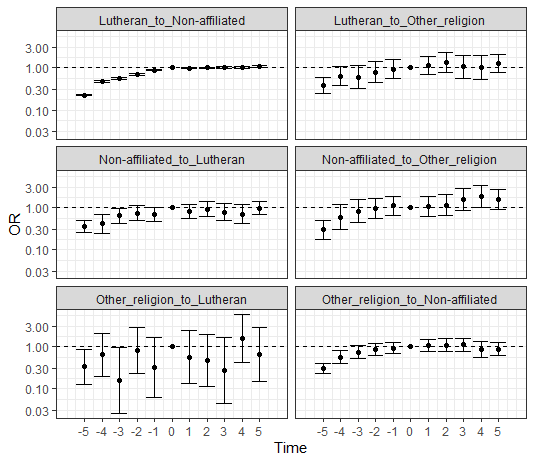


Figure S9. Odds ratios with 95% confidence intervals for mental-health related SA receipt according to time before and after religious switching, by type of switch, for age group 25-37, without control variables included, results from conditional logistic regressions

Notes: Time points -5 and +5 are pooled to include all earlier and later time points, respectively. Odds ratios are presented on a logarithmic scale.


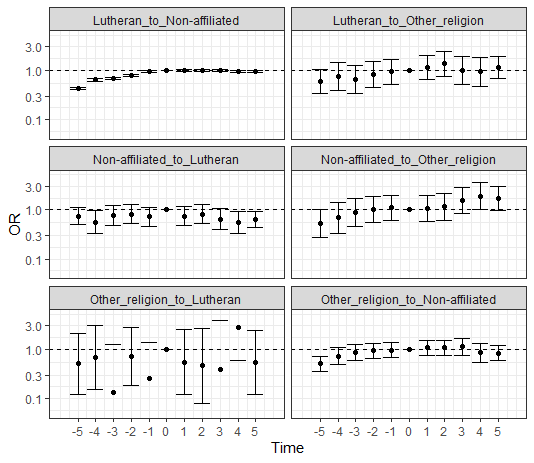


Figure S10. Odds ratios with 95% confidence intervals for mental-health related SA receipt according to time before and after religious switching, by type of switch, for age group 25-37, with control variables included, results from conditional logistic regressions

Notes: Time points -5 and +5 are pooled to include all earlier and later time points, respectively. Odds ratios are presented on a logarithmic scale. Control variables included in the regressions are mother tongue, birth order, birth year, observation year, education, family status, family situation (at age 15), mother’s highest education, father’s highest education, mother’s income quintile (at age 15), father’s income quintile (at age 15), mother’s labour market status (at age 15), father’s labour market status (at age 15), owner occupied dwelling (at age 15), student, and region.

1. https://uefconnect.uef.fi/en/share-fi/ [↑](#footnote-ref-1)
